# Exploring the Impact of Ultrasound-Assisted Extraction on the Phytochemical Composition and Bioactivity of *Tamus communis* L. Fruits

**DOI:** 10.3390/ph18091342

**Published:** 2025-09-06

**Authors:** Irene Gouvinhas, Maria José Saavedra, Maria José Alves, Juliana Garcia

**Affiliations:** 1 Centre for the Research and Technology of Agro-Environmental and Biological Sciences (CITAB), Inov4Agro, University of Trás-os-Montes e Alto Douro, Quinta de Prados, 5000-801 Vila Real, Portugal; 2 AquaValor—Centro de Valorização e Transferência de Tecnologia da Água, Rua Dr. Júlio Martins n.º 1, 5400-342 Chaves, Portugal; 3 LiveWell—Research Centre for Active Living & Wellbeing, Instituto Politécnico de Bragança, 5300-253 Bragança, Portugal; 4 CIMO—Centro de Investigação de Montanha, Instituto Politécnico de Bragança, 5300-253 Bragança, Portugal; 5 Laboratório Associado para a Sustentabilidade e Tecnologia em Regiões de Montanha (SusTEC), Instituto Politécnico de Bragança, Campus de Santa Apolónia, 5300-253 Bragança, Portugal

**Keywords:** *Tamus communis*, ultrasound-assisted extraction, phenolic compounds, antioxidant and anti-inflammatory activity, antimicrobial and anti-aging properties

## Abstract

**Background/Objectives**: The health benefits of *Tamus communis* fruits have been associated with their high phenolic content, which comprises several flavonoids. However, the extraction methods might significantly impact these valuable compounds’ bioactivity. Therefore, the current study assesses how different extraction techniques affect *T. communis* extracts’ antioxidant, anti-aging, antimicrobial, cytotoxic, anti-inflammatory, and phenolic contents. **Methods**: Conventional method (TCE-CM) and ultrasound-assisted extraction (TCE-UM) were the methods employed. **Results**: The increased phenolic content of TCE-UM, particularly flavonoids and phenolic acids, was demonstrated to be a contributing factor to its higher biological activity. Key enzymes linked to dermatological conditions, such as elastase, collagenase, hyaluronidase, and tyrosinase, were significantly inhibited by both extracts at 1 mg/mL; TCE-UM showed the highest tyrosinase inhibition (65.61  ±  5.21%) compared to TCE-CM (21.78  ±  2.19%). TCE-UM also demonstrated exceptional antibacterial performance, showing notable antibiofilm and metabolic inactivation effects and potent activity against pathogens such as *Staphylococcus aureus*, *Escherichia coli*, and *Candida albicans*. Both extracts showed concentration-dependent anti-inflammatory properties; TCE-UM had a lower IC50 value (26.46 ± 2.30%) in nitric oxide inhibition tests, suggesting stronger anti-inflammatory capabilities. **Conclusions**: These findings underscore the superior bioactivity of TCE-UM and suggest that ultrasonic extraction is a more efficient method for isolating bioactive phenolic compounds from *T. communis* fruits, presenting promising applications in anti-aging and antimicrobial formulations.

## 1. Introduction

*Tamus communis* (black bryony) is a wild green plant of the Dioscoreaceae family typically distributed across West, South, and South-Central Europe. It commonly grows in hedgerows, woodland, orchards, and even home gardens. Despite its widespread presence, the fruits are considered toxic to humans, as certain species can cause adverse effects like nasal irritation, oedema, and redness, mainly attributed to the presence of calcium oxalate crystals and triterpene glucosides [1,2].

Despite presenting toxic features, *T. communis* has been traditionally used to treat lumbago, arthrosis, and rheumatism [3]. Recent studies have also highlighted the potential of *T. communis* extracts for topical applications, either through direct use or solvent maceration, as well as their incorporation into hydrogel formulations [4,5]. Anti-inflammatory effects have been reported in localized conditions, including wounds or lesions affecting the subcutaneous and muscle tissues [5]. In addition to these anti-inflammatory effects, other medicinal properties, such as antioxidant and antimicrobial activities [2] have also been suggested, although further validation is still needed.

A growing body of evidence supports that many of these effects are associated with the plant’s high phenolic content, such as gallic acid derivates, and flavonoid compounds, especially apigenin-6-C-glucoside-8-C-glucoside, quercetin-*O*-rhamnosyl-*O*-rhamnoside, and kaempferol-3,4’-di-*O*-rhamnoside, usually identified in *T. communis* extracts [4,5]. Notwithstanding, differences in both the quantity and type of phenolic compounds, as well as the resulting antioxidant properties, might be related to the extraction method used [5].

Current literature highlights that conventional solid–liquid extraction techniques, such as boiling, heating, soxhlet, and cold extraction, have been considered unsuitable for achieving high yields and biological activities since they might involve polyphenols’ thermal degradation and greater solvent expenditure in the extraction process [6]. In this sense, new methods, such as ultrasound-assisted extraction (UAE) and pressurized liquid extraction, have been developed to replace previous methods, promoting a fast, efficient, safe, and energy-saving extraction [6]. UAE technology works on the principle of cavitation, where mechanical waves transfer ultrasonic energy, triggering elevated pressure and leading, thereby, to cavity formation [7]. When cavities cannot absorb more energy, the bubbles collapse, leading to cellular disruption and releasing bioactive compounds [8]. Of note, UAE is considered one of the most efficient and eco-friendly innovative methods to extract intracellular compounds from the cell matrix [9]. This technology allows a higher yield extraction, with minimal solvent usage and reduced time and energy consumption, decreasing polyphenols’ thermal degradation [9].

To the best of our knowledge, no studies concerning the evaluation of *T. communis* phenolic compounds extracted by different methods, and their respective effects in antioxidant and anti-aging activities, have been reported. In this sense, the present work aims to investigate how different extraction techniques (conventional and ultrasound-assisted) influence the phenolic profile of *T. communis* extracts, as well as their biological properties, specifically antioxidant, anti-aging, depigmenting, antimicrobial, antibiofilm, and anti-inflammatory activities. Therefore, while current production is still limited and seasonal, the valorization of *T. communis* fruits, particularly through sustainable extraction strategies, may foster greater interest in their cultivation and support future industrial applications.

## 2. Results and Discussion

### 2.1. Phenolic Composition

**Figure 1:**
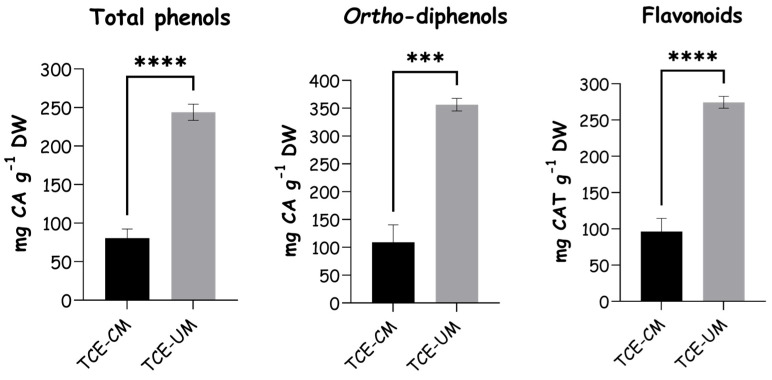
Phenolic content of *T. communis* extracts obtained by ultrasound-assisted extraction (TCE-UM) and traditional solid-to-liquid extraction (TCE-CM). *** and **** indicate statistically significant differences at *p* < 0.001 and *p* < 0.0001, respectively.

The results for phenolic composition, including total phenols, *ortho*-diphenols, and flavonoids, are summarized in Figure 1. Regarding total phenols, after employing ultrasound-assisted extraction (TCE-UM), a significant increase was observed (243.94 ± 8.54 mg CA g^−1^) when compared with traditional solid-to-liquid extraction (TCE-CM), which presented 80.43 ± 9.82 mg CA g^−1^. The results demonstrated that the ultrasound method was more effective in obtaining phenolic compounds from *T. communis*. In fact, TCE-UM yielded 19.4 ± 0.8% *w*/*w* of dry extract relative to dry fruit, whereas the TCE-CM gave 7.6 ± 0.5% *w*/*w*. A similar trend was observed for *ortho*-diphenols once TCE-UM showed a significantly higher content (356.46 ± 9.17 mg CA g^−1^) over TCE-CM (109.02 ± 25.59 mg CA g^−1^). For flavonoids, TCE-UM also demonstrated superior extraction efficiency (274.49 ± 6.59 mg CAT g^−1^) compared to TCE-CM (96.41 ± 14.61 mg CAT g^−1^). This strongly suggests that TCE-UM provided greater amounts of total phenols, *ortho*-diphenols, and flavonoids when compared to the classical extraction process in *T. communis* and conventional techniques. It should be noted that the extraction methods were conducted with different sample-to-solvent ratios (1:1250 for the conventional method and 1:20 for the UAE). This discrepancy resulted from the specific requirements of each protocol: the conventional extraction was based on a small-scale sequential approach, while UAE required a higher mass of plant material to ensure proper sonication. Although both methods used the same solvent composition, this difference in extraction scale may influence the yield and composition of the extracts and should be considered when interpreting the results.

Additionally, while several studies have investigated *T. communis*, most have focused primarily on its roots and rhizomes, with relatively few examining the fruit. This highlights a gap in the literature, as comprehensive studies on the fruit are still limited. Regarding studies on other parts of plant, a 2009 study on *T. communis* roots reported a total phenolic content of 26.55 mg GAE g^−1^ and a flavonoid content of 10.15 mg QE g^−1^, which are significantly lower than the values obtained in our study, regardless of the extraction method used [10]. However, a more recent study from 2022 reported [2] higher total phenolic content for the conventional extraction method in rhizomes, with values of 167 mg GAE g^−1^. Nevertheless, this is still lower than the value obtained using ultrasound extraction in the present study (243.94 ± 8.54 mg CA g^−1^). In terms of flavonoid content, our study shows markedly higher values compared to those reported in the same 2022 study, where rhizomes yielded only 15.6 mg QE g^−1^ dw.

In this sense, the current study, by analyzing the phenolic composition and antioxidant activity of *T. communis* extracts from the fruit, contributes valuable insights that expand the existing knowledge beyond the more commonly studied underground parts.

**Figure 2:**
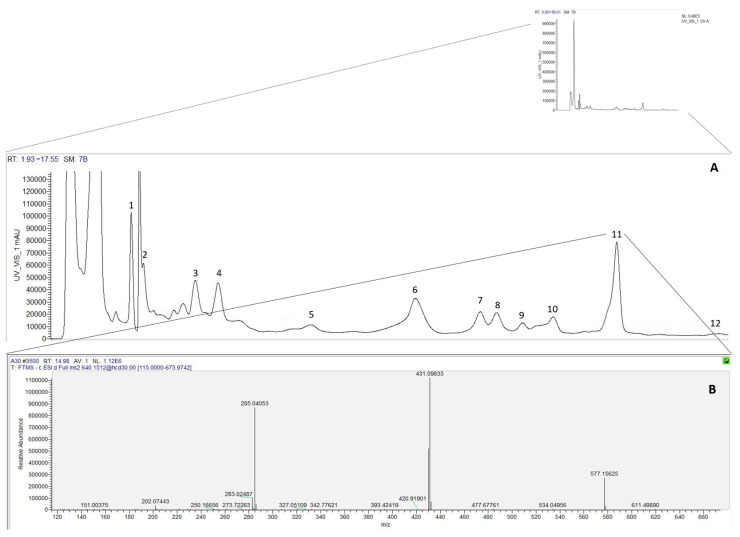
(**A**) HPLC-UV/VIS chromatogram at 330 nm of *Tamus communis* extract obtained by ultrasound-assisted extraction (TCE-UM). (**B**) Mass spectrum of the peak at 14.98 min, identified as kaempferol-3,7-di-*O*-rhamnoside.

In the HPLC–DAD–ESI-MS/MS analysis of *T. communis* (Figure 2), which was extracted by both conventional solid–liquid extraction (TCE-CM) and ultrasound-assisted extraction (TCE-UM), twelve compounds were identified, including phenolic acids, flavonols, and flavan-3-ols (Table 1).

Once again, the ultrasound-assisted methodology has once again proved to be more efficient for phenolic compound extraction, as demonstrated in the table showing the identification and quantification of these compounds by HPLC coupled to mass spectrometry. Except for kaempferol-3-*O*-rutinoside (*p* > 0.05), all compounds demonstrated significantly increased concentrations when the ultrasound method was used over conventional method. In this one, concentrations ranged from trace amounts to 0.90 mg mL^−1^, while in the ultrasound-assisted method, they ranged from 0.01 to 1.17 mg mL^−1^.

The increase in concentrations in most phenolic compounds, analysed by HPLC-MS, indicates a high cell-disruption process and an enhancement in the liberation of bioactive compounds due to ultrasonication. As reported from the present study and earlier literature [19], it is once more evident that this method generally results in the highest levels across a broad range of phenolic compounds, making it an attractive candidate method among all other techniques for maximizing the extraction of valuable phytochemicals from plant materials.

Several studies have also previously analyzed the phenolic profile of *T. communis*, identifying both common and distinct compounds compared to the present study. For instance, some of these studies reported different compounds, such as chlorogenic acid, quercetin rhamnoside hexoside, kaempferol pentoside, and kaempferol hexoside, which were not detected in this analysis. However, consistent with our findings, they also identified a compound, namely kaempferol-*O*-rhamnoside (9.9 µg/g), which was found in both the current and previous research. This overlap highlights the recurrent presence of key flavonoids in *T. communis* across different extraction methods and analytical approaches. In addition, another study by the same authors also analyzed the fruit of *T. communis* [4]. In that study, the common compounds identified were quercetin-3-*O*-neohesperoside (453 µg/g) and kaempferol-3-*O*-neohesperidoside (430.8 µg/g). Moreover, they detected several luteolin and apigenin derivatives, which were not identified in the present work. This highlights differences in the phenolic profiles depending on extraction methods or plant parts analyzed, suggesting the need for further investigation into the complete phenolic composition of *T. communis*.

Although some of the identified compounds were present in trace amounts, their contribution to the overall bioactivity cannot be disregarded. Several studies have shown that even low-abundance phenolic compounds can act synergistically with major components, enhancing the biological effects of complex plant extracts [20,21]. Therefore, the observed bioactivities of *T. communis* extracts may result from the combined and possibly synergistic effects of both major and minor phenolics.

Beyond extraction yield, process-level metrics also highlight the advantages and limitations of ultrasonic-assisted extraction (UAE) when benchmarked against two other widely cited “green” techniques—microwave-assisted extraction (MAE) and supercritical-fluid extraction (SFE). Under the conditions optimised in the present work, UAE operates at sub-ambient pressure and moderate temperatures (<55 °C) with short acoustic pulses, cutting overall energy demand by ≈20–50% versus conventional solvent reflux [22]. MAE reduces residence time even further (<10 min) but does so at the expense of high instantaneous power inputs and elevated bulk temperatures (80–120 °C); a recent orange-peel study still reported ~0.2 kWh of electricity and 160 g CO_2_ eq. per batch, despite the time savings [23]. In contrast, SFE virtually eliminates organic-solvent residues by using scCO_2_ modified with ≤15% ethanol, yet it requires 100–500 bar compression and longer static–dynamic cycles (30–90 min), shifting the energy burden from heating to gas pressurisation and increasing capital cost [24]. Taken together, these figures show that UAE provides a favourable trade-off—minimal solvent, low-to-moderate energy input, and ambient-pressure operation—while avoiding the thermal stress of MAE and the high-pressure infrastructure demanded by SFE.

### 2.2. Antioxidant Capacity Analysis

**Figure 3:**
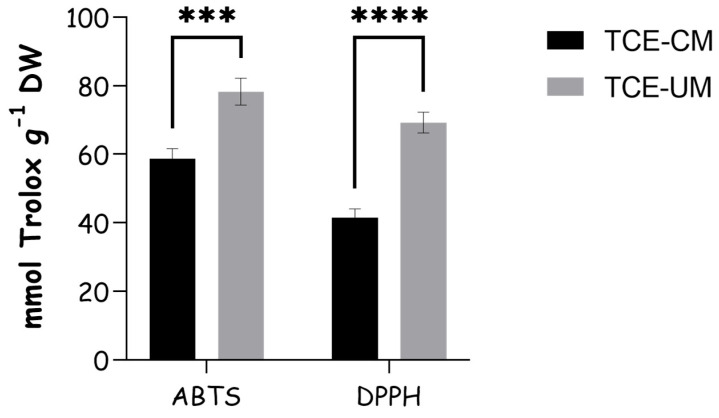
Antioxidant capacity of *T. communis* extracts obtained by ultrasound-assisted extraction (TCE-UM) and traditional solid-to-liquid extraction (TCE-CM). *** and **** indicate statistically significant differences at *p* < 0.001, and *p* < 0.0001, respectively.

Figure 3 shows the antioxidant capacity results obtained by the ABTS and DPPH assays. TCE-UM was more efficient in terms of antioxidant capacity tested by ABTS assay as compared to TCE-CM with the values of 78.28 ± 3.21 and 58.64 ± 2.46 mmol Trolox g^−1^, respectively. With regard to DPPH scavenging capacity, TCE-UM extract showed higher radical scavenging (69.29 ± 2.49 mmol Trolox g^−1^) compared to the TCE-CM extract (41.51 ± 2.06 mmol Trolox g^−1^). This suggests the ultrasound-assisted extraction was more effective in extracting compounds with ABTS and DPPH radical-scavenging activities. The magnitude of the ABTS activity observed for the UAE extract (78.28 ± 3.21 mmol TE g^−1^) greatly surpasses values typically reported for culinary Lamiaceae herbs that are widely regarded as antioxidant benchmarks. For example, an *Origanum vulgare* hydro-ethanolic extract collected in the Chilean Altiplano showed 1.25 mmol TE g^−1^, while *Salvia officinalis* essential oil reached only 0.098 ± 0.005 mmol TE g^−1^ under the same assay. These data underscore the exceptional radical-quenching capacity of *T. communis* fruit when extracted by UAE [25,26]

### 2.3. Skin-Related Enzyme’s Inhibitory Effects

**Figure 4:**
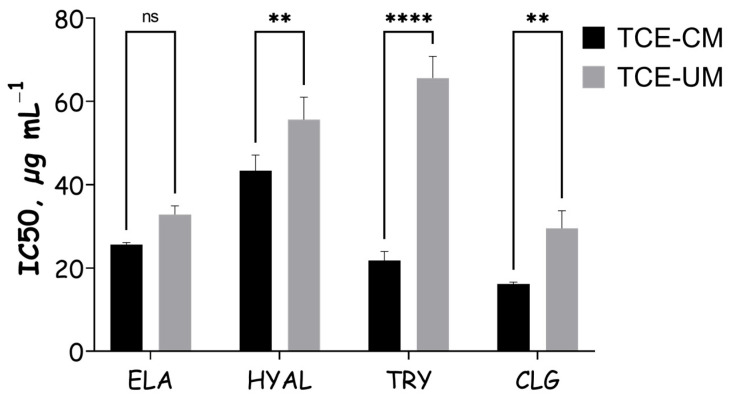
Skin-related enzyme inhibitory effects of *T. communis* extracts. ns indicates non-significant differences; ** and **** indicate statistically significant differences at *p* < 0.01 and *p* < 0.0001, respectively.

This study evaluated the *T. communis* extracts for their ability to inhibit key enzymes related to skin aging and pigment processes (Figure 4). The results indicated that both extracts, particularly TCE-UM, demonstrated significant inhibitory activity across all enzymes tested. To our knowledge, the present study is the first to demonstrate the anti-elastase, anti-hyaluronidase, anti-collagenase, and anti-tyrosinase activities of *T. comnunis* extracts. Tyrosinase was highly sensitive to TCE showing maximum inhibition of more than 60% for TCE-UM. Tyrosinase is a key enzyme involved in melanin production, and its overactivity can lead to hyperpigmentation. The tyrosinase inhibition observed in this study was notably higher in the TCE-UM extract (65.61 ± 5.21%) compared to the TCE-CM extract (21.78 ± 2.19%). This difference in inhibitory activity can be explained by the greater presence of bioactive compounds in the TCE-UM extract, as ultrasonic extraction typically enhances the yield of phenolic compounds such as flavonoids, as previously observed. Among these compounds, kaempferol, quercetin, and apigenin are known for their significant tyrosinase inhibitory activities [27]. A study demonstrated that apigenin, commonly found in plant extracts, exhibited strong tyrosinase inhibition [28]. Similarly, kaempferol and quercetin have been shown to inhibit the oxidation of L-DOPA catalyzed by mushroom tyrosinase, with their inhibitory activity likely stemming from their copper-chelating ability. The higher concentration of these compounds in the TCE-UM extract could explain its stronger inhibitory effect on tyrosinase, as copper chelation plays a crucial role in enzyme inhibition. Thus, these bioactive compounds, particularly in higher amounts in the TCE-UM extract, likely contribute to its superior tyrosinase inhibition activity.

The elastase inhibition percentage for the TCE-UM extract was significantly higher (36.17 ± 6.26%) compared to TCE-CM (25.61 ± 0.53%), while collagenase inhibition was also more potent in TCE-UM (26.17 ± 6.26%) versus TCE-CM (16.12 ± 0.47%). These results indicate that the ultrasound-assisted extraction method enhances the inhibitory activity against these enzymes, potentially through a higher yield of bioactive compounds. This suggests that phenolic compounds, known for their enzyme-inhibiting capabilities, are more efficiently extracted by ultrasound, leading to increased enzyme inhibition. The individual polyphenols identified in both extracts may contribute differently to inhibiting elastase and collagenase activities. Phenolic acids like caffeic acid hexoside and 3-*O*-caffeoylquinic acid have been reported to form non-covalent interactions with elastase and collagenase, potentially inhibiting these enzymes through hydrogen bonding with side chain groups. This could explain the enhanced inhibition of elastase and collagenase by the TCE-UM extract, which had higher concentrations of these specific phenolic compounds. Notably, phenolic acids are known for their ability to interact with zinc ions in the active site of collagenase, further contributing to the inhibition of this enzyme [29]. This interaction could explain the significant difference in collagenase inhibition between TCE-UM and TCE-CM. The enhanced elastase inhibition observed in TCE-UM may be linked to its higher flavonoid content, as flavonoids are known to inhibit elastase through their interaction with the enzyme’s active site, leading to competitive inhibition [29].

The ultrasound-assisted extraction led to significantly higher hyaluronidase inhibition compared (55.61 ± 5.39%) to the conventional method (43.30 ± 3.84%), which can be attributed to the increased concentration of phenolic compounds in TCE-UM. The enhanced inhibitory activity is likely due to the structural characteristics of the polyphenols, including the presence of galloyl and *ortho*-dihydroxyphenol groups, which are known to interact strongly with the enzyme’s active site. These findings align with previous studies showing that polyphenols with multiple hydroxyl groups, particularly those containing galloyl units, exhibit potent hyaluronidase inhibitory activity [30]. When benchmarked against other polyphenol-rich botanicals, the multi-enzyme profile of the UAE fraction is highly competitive. A survey of 23 commercial plant extracts found that green-tea preparations inhibited elastase by ≈10% and collagenase by ≈47% at comparable loadings, values notably lower (elastase) or only modestly higher (collagenase) than the 36% and 26% we report here for *T. communis* UAE. Likewise, licorice-root extract, widely used as a depigmenting agent, exhibits a tyrosinase IC_50_ of 34.5 µg mL^−1^, whereas our UAE extract achieves >60% inhibition at 200 µg mL^−1^. These comparisons reinforce the view that the *T. communis* fruit extract delivers a favourable balance of broad-spectrum enzyme inhibition relative to established phytocosmetic standards [31].

Structure-activity studies show that hydroxycinnamic acids, such as the caffeic- and sinapic-acid derivatives present in our extract, can inhibit collagenase and elastase chiefly by chelating the catalytic Zn^2+^ and stabilising the complex with multiple hydrogen bonds [31,32,33]. Conversely, flavone and flavonol glycosides detected here (apigenin-C-diglucoside, quercetin- and kaempferol-neohesperosides) bind to tyrosinase and hyaluronidase via π–π stacking with the conserved catalytic histidines together with an extensive H-bond network, and generally display higher predicted affinities [33]. These literature trends support the qualitative hierarchy observed experimentally—flavonol ≈ flavone > hydroxycinnamate for tyrosinase; hydroxycinnamate ≈ flavonol for elastase; flavone for hyaluronidase; hydroxycinnamate ≥ flavonol for collagenase, suggesting that metal chelation and aromatic π interactions underpin the broad-spectrum inhibition reported in Table 2.

### 2.4. Antimicrobial Activity

The antimicrobial activity of the TCE-CM and TCE-UM extracts was assessed against both multidrug-resistant clinical isolates and foodborne pathogens (Table 2). The MIC (Minimum Inhibitory Concentration) and MBC (Minimum Bactericidal Concentration) values indicated that both extracts exhibited varying degrees of efficacy across the tested organisms. For *S. aureus*, TCE-UM demonstrated stronger inhibitory activity with a MIC of 31.25 µg/mL compared to TCE-CM (62.5 µg/mL), although both extracts showed bacteriostatic rather than bactericidal effects as the MBC was greater than the highest concentration tested (>31.25 µg/mL for TCE-UM and >62.5 µg/mL for TCE-CM). In the case of *E. coli*, TCE-UM also displayed a significantly lower MIC of 125 µg/mL, compared to TCE-CM’s 500 µg/mL, again with both extracts requiring concentrations beyond the maximum tested for bactericidal action. For *P. aeruginosa* and *A. baumannii*, both extracts showed limited inhibition, with MIC values of >1000 µg/mL for TCE-CM and 1000 µg/mL for TCE-UM against *P. aeruginosa*, while for *A. baumannii*, TCE-UM showed a slightly enhanced MIC of 500 µg/mL compared to TCE-CM’s 1000 µg/mL. The yeast *C. albicans* was more susceptible to both extracts, with TCE-UM having a MIC and MBC of 62.5 µg/mL, while TCE-CM displayed a MIC of 250 µg/mL and an equivalent MBC. Among the foodborne isolates, *Y. enterocolitica* was the most susceptible, with MIC values of 125 µg/mL and 500 µg/mL for TCE-UM and TCE-CM, respectively, corresponding to their MBC values. The results demonstrated that TCE-UM exhibited superior antimicrobial activity in most cases, particularly against *S. aureus*, *E. coli*, *K. pneumoniae*, and *C. albicans*. This enhanced activity could potentially be attributed to the higher concentration of phenolic compounds in the TCE-UM extract. Phenolic compounds, known for their antimicrobial properties, may inhibit bacterial growth through various mechanisms, such as disrupting cell membranes and interfering with enzyme activity. The greater presence of these bioactive compounds in TCE-UM likely contributed to its more potent inhibitory effects across the tested organisms.

### 2.5. Biofilm Removal

The antibiofilm activity was assessed exclusively for the TCE-UM extract, as this extract demonstrated superior antimicrobial activity compared to other tested samples. The evaluation of biofilm removal was carried out using the crystal violet assay across various concentrations. TCE-UM showed significant efficacy in reducing biofilm formation for the tested organisms. For *C. albicans*, biofilm biomass significantly decreased at 1 × MIC (19.6 ± 0.5%) and continued to reduce with increasing concentrations, reaching a marked reduction at 5 × MIC (55.3 ± 1.7%) and a substantial effect at 10 × MIC (76.7 ± 7.3%). In the case of *E. coli*, the biofilm removal was more pronounced at 5 × MIC (39.7 ± 1.1%) and further increased at 10 × MIC (62.4 ± 9.9%). Similarly, for *S. aureus*, biofilm removal was initially moderate at 1 × MIC (12.8 ± 1.7%) but improved significantly at 10 × MIC (85.8 ± 4.5%). For *K. pneumoniae*, there was a noticeable enhancement in biofilm removal from 1 × MIC (21.7 ± 3.3%) to 10 × MIC (83.7 ± 2.3%). *A. baumannii* also exhibited a considerable reduction in biofilm formation at higher concentrations, peaking at 10 × MIC (41.6 ± 7.0%). The biofilm removal efficacy of TCE-UM against *P. aeruginosa* showed steady improvement across the concentrations, reaching a significant reduction at 10 × MIC (42.8 ± 3.3%). In conclusion, TCE-UM extract demonstrated a concentration-dependent biofilm removal activity across all tested microorganisms, with notable effects, particularly at higher concentrations. Further investigation is needed to elucidate the exact mechanisms behind the observed efficacy of TCE-UM, particularly against resistant strains like *P. aeruginosa* and *A. baumannii*, which are typically challenging to treat.

### 2.6. Metabolic Inactivation Results

**Figure 5:**
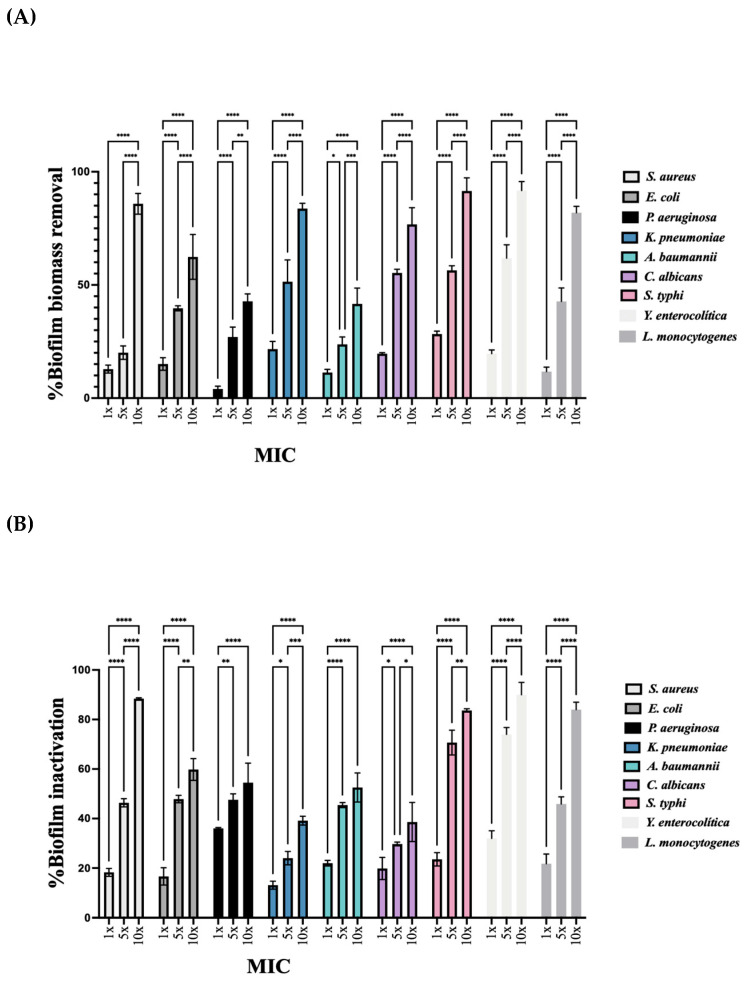
Effect of TCE-UM Extract on Biofilm Biomass Removal and Metabolic Inactivation. Effect of TCE-UM extract at MIC, 5 × MIC, and 10 × MIC on biofilms of various microorganisms. (**A**) Percentage of biofilm biomass removal, (**B**) Percentage of biofilm metabolic inactivation. Mean values ± SD for three independent experiments are shown. Both biofilm biomass removal and metabolic inactivation show a concentration-dependent increase across all tested organisms, with the highest effects observed at 10 × MIC. *, **, ***, and **** indicate statistically significant differences at *p* < 0.05, *p* < 0.01, *p* < 0.001, and *p* < 0.0001, respectively.

The metabolic inactivation of biofilms for TCE-UM extract was assessed using a resazurin assay, as shown in Figure 5. The results revealed a concentration-dependent inhibition across the tested microorganisms. For *S. aureus*, at 1 × MIC, the metabolic inactivation was moderate (18.2 ± 1.6%) but increased significantly at 5 × MIC (46.4 ± 1.6%), reaching maximum inhibition at 10 × MIC (88.4 ± 0.3%).

In the case of *E. coli*, the biofilm’s metabolic activity was more effectively reduced at 5 × MIC (47.9 ± 1.5%), with a further increase at 10 × MIC (59.8 ± 4.4%). *P. aeruginosa* showed a similar trend, with initial inhibition at 1 × MIC (35.9 ± 0.3%), improving at 5 × MIC (47.5 ± 2.4%), and peaking at 10 × MIC (54.4 ± 7.8%). For *K. pneumoniae*, the metabolic inactivation remained relatively low at 1 × MIC (13.1 ± 1.6), but increased at higher concentrations, particularly at 10 × MIC (39.1 ± 1.8%). Similarly, *A. baumannii* exhibited increased inactivation with higher concentrations, from 21.9 ± 1.1% at 1 × MIC to 52.5 ± 5.8% at 10 × MIC. Regarding *C. albicans*, at 1 × MIC, the metabolic activity reduction was moderate (19.8 ± 4.4%) but increased significantly at 5 × MIC (29.7 ± 0.7%) and reached substantial inhibition at 10 × MIC (38.5 ± 7.9%). In the case of *S. typhi* and *Y. enterocolitica*, there was a clear increase in metabolic inactivation as the concentration increased. For *S. typhi*, at 1 × MIC, the inhibition was 23.5 ± 2.7%, increasing to 70.7 ± 4.9% at 5 × MIC and reaching 83.6 ± 0.6% at 10 × MIC. *Y. enterocolitica* followed a similar pattern, with 31.9 ± 3.0% at 1 × MIC, increasing to 73.9 ± 2.7% at 5 × MIC and peaking at 89.9 ± 4.9% at 10 × MIC. Lastly, for *L. monocytogenes*, the metabolic inactivation was moderate at 1 × MIC (21.7 ± 3.8%) but improved notably at 5 × MIC (45.8 ± 2.8%), reaching significant inhibition at 10 × MIC (83.9 ± 2.9%).

The results showed that the TCE-UM extract exhibited concentration-dependent metabolic inactivation across all tested microorganisms, with the highest biofilm inhibition occurring at 10 × MIC.

### 2.7. Cell Toxicity and Anti-Inflammatory Activity of Tamus communis Extracts

**Figure 6:**
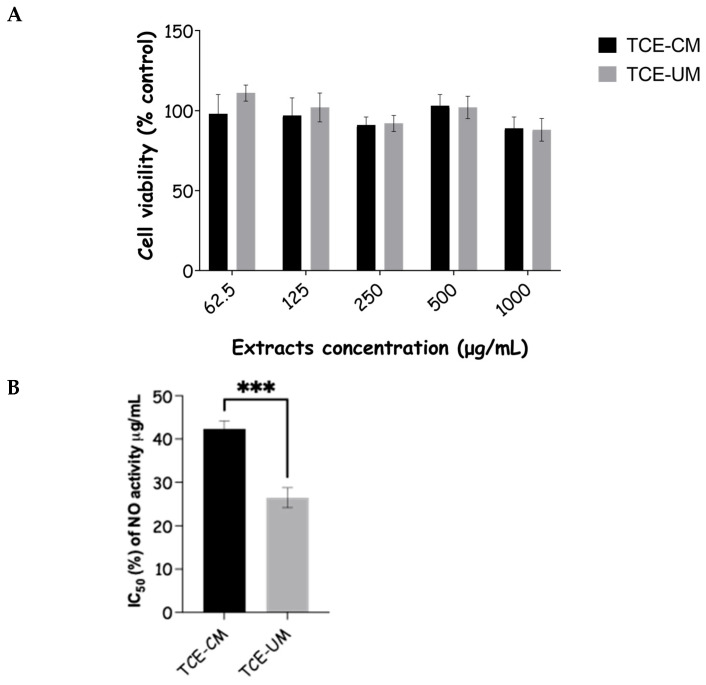
The effects of *T. communis* extracts on the viability of RAW264.7 macrophage cells (**A**) and levels of NO (**B**). Data represent the mean values of three experiments (± SD). *** indicate statistically significant differences at *p* < 0.001.

Before evaluating the anti-inflammatory activity of *T. communis* extracts, cell toxicity assays were conducted to ensure that the concentrations used would not compromise cell viability and, therefore, the accuracy of the anti-inflammatory results. The results revealed that cell viability remained above 90% at all concentrations tested, indicating that neither extract exhibited significant cytotoxic effects. The high cell viability across all tested concentrations allowed the safe application of these extracts for subsequent anti-inflammatory assays without the risk of toxicity-induced artifacts. This finding suggests that *T. communis* extracts are well-tolerated by cells within the tested concentration range, thus establishing a robust baseline for their anti-inflammatory potential. Following the confirmation of non-toxicity, the anti-inflammatory activity of *T. communis* extracts was assessed by measuring the inhibition of nitric oxide (NO) production in lipopolysaccharide (LPS)-stimulated RAW 264.7 macrophages (Figure 6). The LPS stimulation induces the overproduction of NO, a key inflammatory mediator, via upregulation of inducible nitric oxide synthase (iNOS). The anti-inflammatory activity was quantified as the percentage inhibition of NO production, with IC50 values of 42.36 ± 1.84% for the TCE-CM extract and 26.46 ± 2.30% for the TCE-UM extract. Both extracts significantly reduced NO production in a concentration-dependent manner, demonstrating potent anti-inflammatory effects when compared to the control group, where NO production was 100% in the LPS-stimulated macrophages.

The greater anti-inflammatory effect observed in the ultrasound-assisted extract (TCE-UM), as evidenced by its lower IC_50_ value, suggests that this extraction method may yield a higher concentration of bioactive polyphenolic compounds, enhancing its inhibitory potential against NO production. This finding aligns with previous studies where polyphenol-rich extracts, particularly those containing compounds like flavonoids, tannins, and phenolic acids, were shown to inhibit NO production by downregulating pro-inflammatory enzymes such as iNOS and cyclooxygenase-2 (COX-2). The phenolic composition of *T. communis* extracts, similar to that which was previously associated with elastase and collagenase inhibition, is likely responsible for their anti-inflammatory effects. Polyphenols, particularly those with galloyl or catechol groups, are known to suppress the production of pro-inflammatory mediators such as NO, TNF-α, and interleukins (IL-1β, IL-6), which LPS stimulates in macrophages [34]. This inhibition is thought to occur through the modulation of key signaling pathways involved in the inflammatory response.

## 3. Materials and Methods

### 3.1. Chemicals

The radical compounds 2,2-diphenyl-1-picrylhydrazyl (DPPH•) and 2,2′-azino-bis(3-ethylbenzothiazoline-6-sulfonic acid) diammonium salt (ABTS•+), as well as 6-hydroxy-2,5,7,8-tetramethylchroman-2-carboxylic acid (Trolox), potassium persulfate, and the enzymes tyrosinase and elastase, along with other reagents for the enzymatic assays, were obtained from Sigma-Aldrich (Steinheim, Germany). Methanol, saline solution (0.9% NaCl), and sulfanilamide were supplied by Merck (Darmstadt, Germany). Microbiological culture media and antibiotics used in the antimicrobial assays were purchased from Oxoid (Thermo Fisher Scientific Inc., Basingstoke, UK). Ultrapure water was produced with a Millipore purification system (Merck Millipore, Burlington, MA, USA). Hydrochloric acid was sourced from Fluka Chemika (Neu-Ulm, Germany). Cell culture reagents, including Dulbecco’s Modified Eagle Medium (DMEM), fetal bovine serum (FBS), penicillin, streptomycin, and Alamar Blue^®^ (AB) reagent, were acquired from Invitrogen (distributed by Alfagene, Lisbon, Portugal).

### 3.2. Sampling

Fruits of *Tamus communis* were collected in the Mirandela region, located in Trás-os-Montes, northeastern Portugal, during the summer of 2023. The plant material was randomly harvested from multiple individuals within a defined area. Identification was performed based on morphological characteristics following the taxonomic descriptions provided in *Flora Iberica* (Castroviejo, 2001). Voucher specimens (ETBO33, ETBO36, and ETBO53) have been preserved in the herbarium of the University of Trás-os-Montes and Alto Douro (UTAD), Portugal. Each fruit sample was lyophilized (Ly-8-FM-ULE, Snijders, Tilburg, The Netherlands), grounded to a very fine and homogeneous powder using a coffee grinder. Although particle size was not formally measured, the visual uniformity was considered suitable for extraction. The samples were then stored in the deep-freezer at −20 °C for subsequent analyses.

### 3.3. Tamus communis Extracts Preparation

#### 3.3.1. *Tamus communis* Extract-Conventional Method (TCE-CM)

For the preparation of the extract, the lyophilized samples of *T. communis* were ground to a very fine powder (using a coffee mill) and extracted using three-step sequential extraction, with some modifications [35]. Briefly, to the plant material (40 mg), 50 mL of an 70% ethanol solution (*v*/*v*, in water) were added. The mixture was agitated for 1 h (orbital shaker, 150 rpm, room temperature) and then centrifuged (10,000 rpm, 4°C; for 5 min (Sigma Centrifuges 3–30 K, St. Louis, MO, USA)). The supernatant was filtered and collected, followed by the addition of another 50 mL of 80% ethanol to the pellet. This procedure was repeated 3 times for each sample of *T. communis*. This sequential extraction resulted in an approximate sample-to-solvent ratio of 1:1250. Supernatants were filtered through a 0.45-µm PVDF filter (Millex HV13, Millipore, Bedford, MA, USA) and concentrated in a rotary evaporator (35 °C), to remove the ethanol. Extracts were then lyophilized, weighed to calculate the yields and stored at 4 °C for subsequent analysis.

#### 3.3.2. *Tamus communis* Extract—Ultrasound-Assisted Method (TCE-UM)

The protocol used for phenolic extraction was carried out as previously reported with an ultrasonic apparatus (VCX 500 Vibra-CellTM, Newtown, CT, USA) [36] equipped with a 13 mm-diameter tip, operating at 50% amplitude (≈250 W output power) and a constant temperature of 70 °C for 40 min. The ultrasound apparatus was used to extract phenolic compounds from powered samples (2.5 g) with 50 mL of ethanol: water solution (70:30 *v*/*v*), using a sample-to-solvent ratio of 1:20, as required for efficient sonication with the equipment used. Hereafter, the ethanolic extracts were centrifugated (Sigma Centrifuges 2–16 K, Oberkochen, Germany) at 15,493× *g*, for 15 min at 4 °C and filtered. Afterward, the extraction solvent was also evaporated, then lyophilized, weighed to calculate the yields and stored at 4 °C for subsequent analysis.

### 3.4. Phenolic Composition

The quantification of total phenols, *ortho*-diphenols, and flavonoids were determined using spectrophotometric techniques based on previously established protocols [35]. All assays were conducted in triplicate using 96-well microplates (Nunc, Roskilde, Denmark). Absorbance readings were obtained with a microplate reader (Infinite M200, Tecan, Grodig, Austria).

Folin–Ciocalteu spectrophotometric method was performed using caffeic acid (CA) as standard to evaluate the *T. communis* total phenolic content. To determine the content, the Folin–Ciocalteu reagent and sodium carbonate were added to either the CA standard or the sample. Absorbance was measured at 725 nm, and results were expressed as milligrams of caffeic acid per gram of dry weight (mg CA g^−1^ DW). For determination of *ortho*-diphenol content, sodium molybdate and caffeic acid or sample were mixed. Absorbance was measured at 370 nm, using caffeic acid (CA) as the calibration standard. Results were expressed as milligrams of CA per gram of dry weight (mg CA g^−1^ DW).

The total flavonoid content in *T. communis* was assessed using the aluminum chloride colorimetric method, with catechin as the reference compound. Absorbance was recorded at 510 nm, and the values were expressed as milligrams of catechin per gram of dry weight (mg CAT g^−1^ DW).4.4.1. Individual phenolic compounds

The analyses were carried out using HPLC-DAD-ESI-MS/MS (High Performance Liquid Chromatography—Diode Array Detector–tandem Mass Spectrometry, Thermo Scientific, Waltham, MA, USA). Mass spectrometric detection was carried out on a triple quadrupole mass spectrometer (TSQ Quantum Access Max, Thermo Scientific, Waltham, MA, USA) equipped with an electrospray ionization (ESI) source. Chromatographic separation was achieved using an ODS Hypersil C18 analytical column (250 mm × 4.6 mm, 5 μm particle size, 120 Å pore size, fully porous type A silica). Samples (20 µL) were injected and eluted using a gradient composed of solvent A (0.1% formic acid in water) and solvent B (methanol). The gradient started with 100% A, reached 95% at 22 min, maintained 95% until 25 min, and returned to 100% A at 30 min. The solvent flow rate was set to 0.7 mL/min, and the column temperature was set at 30 °C. Mass spectrometry detection conditions were as follows: capillary temperature at 300 °C, sheath gas pressure at 30 psi, and spray voltage set to 4.0 kV in positive scan mode and 2.5 kV in negative scan mode. The standard solutions were also separately infused to the mass spectrometer, and MS fragment ions were obtained. The deprotonated molecular ions [M − H]^−^ of these compounds were found to be stable in the full scan mass spectra with higher peaks (Table 1). Identification of the phenolic compounds was based on neutral loss scan data for these ions, interpretation of collision-induced dissociation fragments, retention time data, and comparison with literature data.

### 3.5. Antioxidant Capacity

The antioxidant activity was determined through ABTS•^+^ and DPPH radical scavenging assays, as described by Taghouti et al. 2020 [35]. In the ABTS•^+^ assay, absorbance was recorded at 734 nm after 15 min of reaction, while for the DPPH assay, absorbance was measured at 520 nm after the same reaction time. The tests were performed using microplate reader infinite M200 (Tecan, Grodig, Austria) and the scavenging potential was expressed as millimoles of Trolox per gram of dry weight (mmol T/g DW).

### 3.6. Anti-Aging Capacity

Anti-aging capacity was assessed through the inhibition of elastase, collagenase, and hyaluronidase enzymes. All extracts were tested at a concentration of 1 mg/mL in these assays. The elastase and hyaluronidase were evaluated according to our previous studies [37].

Collagenase inhibition was carried out according to Nicolaus et al., 2017. The assay was conducted in a 96-well microplate format using an Infinite F200 Pro microplate reader (Tecan, Männedorf, Switzerland), equipped with filter-based technology [38]. The reaction was tracked using an excitation wavelength of 320 nm and emission at 400 nm, with phosphoramidon employed as a positive control. The total reaction volume of 100 μL consisted of 25 μL of substrate, 25 μL of enzyme, 25 μL of buffer solution, and 25 μL of sample or positive controls. Both enzyme and substrate were prepared in a reagent buffer (10 mM Tris–HCl, pH 7.3) and diluted from stock solutions to final concentrations of 100 μg/mL for the enzyme and 55.55 μg/mL for the substrate, freshly prepared prior to use. Buffer and sample solutions were added first, followed by the enzyme, and the mixture was incubated for 10 min. The reaction was initiated by adding the fluorogenic substrate, and fluorescence was measured after 30 min, with comparisons made to the blank control. A negative control was prepared by replacing the test substance with 25 μL of 10 mM Tris–HCl buffer. Both TCE-CM and TCE-UM extracts were tested in triplicate. Moreover, a blank control was included for each sample concentration to avoid false-positive results caused by any inherent fluorescence from the tested compounds. The percentage of enzyme inhibition was determined using the formula: enzyme inhibition activity (%) = [(FU control − FU sample)/FU control] × 100, where FU represents fluorescence units.

### 3.7. Depigmenting and Lightening Properties

The skin whitening activity was assessed by inhibition of tyrosinase according to our previous study [36]. The extracts were tested at a final concentration of 1 mg/mL.

### 3.8. Antimicrobial Capacity Screening

#### Microorganisms and Culture Media

The microorganisms used were clinical isolates from patients hospitalized in various departments of the Hospital Center of Trás-os-Montes and Alto Douro (CHTMAD). The Ethics Committee of CHTMAD granted this study’s ethical approval, under a collaborative research agreement established in 2004. The isolates, part of the MJH and MJMC collections, are preserved at −70 °C in BHI broth supplemented with 15% (*v*/*v*) glycerol and maintained at the Medical Microbiology Laboratory (Antimicrobials, Biocides, and Biofilms Unit) within the Department of Veterinary Sciences at the University of Trás-os-Montes and Alto Douro (UTAD). The extracts were tested against several strains, including wound exudate-derived pathogens (*Staphylococcus aureus*, *Escherichia coli*, *Pseudomonas aeruginosa*, *Klebsiella pneumoniae*, *Acinetobacter baumannii*, *Candida albicans)* and food-poisoning bacterial strains (*Salmonella typhi*, *Yersinia enterocolitica*, *Listeria monocytogenes*). Identification of these strains was performed based on colony morphology, Gram staining, standard biochemical procedures, and MicroScan WalkAway system panels. Antimicrobial susceptibility profiles were further assessed using the Kirby–Bauer disk diffusion method with various antibiotics.

### 3.9. Antimicrobial Activity

The minimum inhibitory concentration (MIC) was determined using the resazurin microdilution assay [39]. Bacterial strains were collected from overnight cultures grown in Brain Heart Infusion (BHI) broth. A small portion of bacteria was transferred into a bottle with 50 mL of Mueller Hinton Broth (MHB), capped, and placed in an incubator overnight at 37 °C. Following 16 h of incubation, bacterial suspensions were adjusted to an optical density of 0.5 at 500 nm. Resazurin solution (3.4 mg/mL) was freshly prepared in sterile distilled water. A sterilized 96-well microplate was used for the assay. Each well received 100 μL of Mueller–Hinton Broth (MHB) and 200 μL of the extract or positive control. A two-fold serial dilution was performed along each row by transferring 100 μL from one well to the next, covering concentrations from 1000 to 7.81 μg/mL. Subsequently, 20 μL of the bacterial suspension and 20 μL of the resazurin solution were added to each well. Plates were incubated at 37 °C for 18–24 h. All experiments were conducted in triplicate. MIC values were determined based on the resazurin color shift from blue to pink, indicating microbial growth. For MBC determination (defined as the lowest concentration achieving ≥99% bacterial killing), contents from non-colored wells were plated onto Mueller–Hinton Agar and incubated at 37 °C for 24 h. The lowest concentration showing no visible colony growth was recorded as the MBC.4.10. Antibiofilm activity

#### 3.9.1. Bacterial Adhesion/Biofilm Formation and Exposure to Extracts

The microtiter biofilm assay was used to assess the ability of the *T. communis* extracts to control adhered cells of microorganism biofilms. For this, 96-well polystyrene plates were inoculated with 200 μL of a bacterial suspension adjusted to an OD_620_ nm of 0.04 ± 0.02 and incubated at 37 °C with shaking (150 rpm) for 24 h to allow biofilm formation. After incubation, wells were gently aspirated and rinsed once with 200 μL of sterile 0.85% (*w*/*v*) saline solution to remove non-adherent cells. Subsequently, 180 μL of fresh Mueller–Hinton broth and 20 μL of extract solution were added to each well to achieve final concentrations corresponding to MIC, 5 × MIC, and 10 × MIC. These higher concentrations were chosen based on the known increased resistance of biofilm-embedded cells compared to planktonic forms. After a further 24 h of incubation under the same conditions, the effects of the extracts were assessed by analyzing both biofilm biomass and metabolic activity [39].

#### 3.9.2. Biomass Quantification

Following treatment with the various extracts, the wells of the microtiter plates were emptied and rinsed with 250 μL of sterile 0.85% (*w*/*v*) saline solution to remove non-adherent and weakly adherent bacteria. To fix the remaining biofilm-associated bacteria, 250 μL of 96% (*v*/*v*) ethanol were added to each well and left to act for 15 min, after which the wells were again emptied. Subsequently, 200 μL of a 1% crystal violet solution (Merck, Portugal) were applied to each well and left at room temperature for 5 min to stain the adhered biomass. Excess dye was carefully removed, and 200 μL of 33% (*v*/*v*) glacial acetic acid (Fisher Scientific, Loughborough, UK) were used to dissolve the bound crystal violet. Biofilm biomass was then quantified by measuring the absorbance at 570 nm using a microplate reader. Results were expressed as the percentage reduction in biomass relative to untreated control biofilms (Equation (1)).

%BR = (ODc − ODw)/ODc × 100(1)

where %BR is the percentage of biomass reduction, ODc is the OD570 nm value of control wells, and ODw is the OD570 nm value for the extract-treated wells [39].

#### 3.9.3. Metabolic Activity Quantification

After exposing the pre-formed biofilms to *T. communis* extracts, the wells were carefully aspirated and rinsed with 250 μL of 0.85% (*w*/*v*) sterile saline solution to eliminate non-adherent and loosely attached cells. To evaluate metabolic activity, 190 μL of fresh Mueller-Hinton broth and 10 μL of a 400 μM resazurin solution were then added to each wel. Then, the microtiter plates were incubated for 20 min in the dark at room temperature. Metabolic activity was quantified by measuring the fluorescence at 570 and 590 nm, using a microtiter plate reader. The extent of metabolic inactivation was expressed as a percentage relative to untreated biofilms, serving as the control (Equation (2)).

%MI = (Fluoc − Fluow)/Fluoc × 100(2)

where %MI is the percentage of metabolic inactivation, Fluoc represents the fluorescence intensity of untreated biofilms, and Fluow corresponds to the fluorescence of extract-treated biofilms [39].

### 3.10. Anti-Inflammatory Activity In Vitro

RAW 264.7 macrophages were cultured in 175 cm^2^ flasks with DMEM containing 5% FBS, supplemented with 1% antibiotics and 1% glutamine [40]. The cell line was maintained at 37 °C in a 5% CO_2_ atmosphere, replacing the medium every 3–4 days. Once cells reached confluence, they were detached using a rubber policeman instead of trypsin to avoid removing membrane-bound receptors such as RAGE. The cell suspension was concentrated by centrifugation at 900 rpm for 3 min, then resuspended in a small volume of fresh DMEM with 1% antibiotics and 5% FBS. Cell densities were determined using a Neubauer counting chamber and adjusted with DMEM to obtain 75,000 cells/well when 100 µL of the cell suspension was dispensed into the inner 60 wells of 96-well plates. Sterile distilled water was added to the outer row of wells, and the plates were incubated at 37 °C with 5% CO_2_ for 12 h. Conditioned medium was then replaced with fresh serum-free medium in each well. For assays with extracts, 50 µL of the dilutions (in water) were added one hour prior to the addition of the activator. To ensure consistent cell activation, a combination of 25 µg/mL LPS and 10 U/mL IFN-γ in DMEM was used. Extracts were tested at a maximum concentration of 1 mg/mL, and 0.5 mg/mL. Cells were incubated for 24 h at 37 °C and 5% CO_2_. Media alone was the negative control, while activated cells were the positive control.

### 3.11. Determination of Nitric Oxide Production by Griess Assay

Nitric oxide production was indirectly assessed by measuring nitrite levels, a stable end-product of NO oxidation, using the Griess assay. The reagent was freshly prepared by combining equal parts of 1% sulfanilamide and 0.1% N-(1-naphthyl)ethylenediamine dihydrochloride in 5% hydrochloric acid. Upon reaction with nitrite, a violet-colored complex forms. An aliquot of 70 µL from each well’s supernatant was transferred to a new 96-well microplate and mixed with 70 µL of Griess reagent. Absorbance was then measured at 540 nm. 

### 3.12. Cell Culture and Cell Viability Assay

RAW264.7 murine macrophage cells (ATCC, USA) were cultured in DMEM supplemented with 10% FBS and 1% penicillin-streptomycin, incubated at 37 °C in a 5% CO_2_ atmosphere [40]. Cell viability was assessed using the MTT assay. RAW264.7 cells were treated with 100 μM of pure compounds and reference substances, indomethacin and L-NAME. After 24 h of incubation, MTT was added to each well at a final concentration of 0.5 mg/mL, followed by a 2 h incubation at 37 °C. Post-incubation, the medium was removed, and 100 μL of isopropanol was added to dissolve the formazan crystals. Absorbance was measured at 540 nm using a UV-spectrophotometric plate reader. Cell viability was calculated as the percentage ratio of absorbance in treated cells to that of untreated cells, with all experiments conducted in triplicate.

### 3.13. Statistical Analysis

The phenolic composition, antioxidant, and anti-aging activities data were subjected to the IBM SPSS 22.0 statistical software (SPSS Inc., Chicago, IL, USA). The normality of the distribution was checked using the Shapiro–Wilk test, followed by an assessment of homogeneity of variance with Levene’s test. After confirming these assumptions, a t-Student test was performed to compare the means between the groups, with statistical significance set at *p* < 0.05. All sample results are expressed as mean ± standard deviation (n = 3). Data related to cell viability and anti-inflammatory activity were analyzed using GraphPad Prism 8 software (GraphPad Software, San Diego, CA, USA). Statistical comparisons were performed through one-way and two-way ANOVA, followed by Tukey’s multiple comparison test, considering differences statistically significant at *p* < 0.05.

## 4. Conclusions

The present study shows the significant potential of *Tamus communis* fruit extracts, particularly those obtained using TCE-UM, as a source of bioactive compounds with diverse biological activities. The results reveal that TCE-UM is more effective than TCE-CM in extracting phenolic compounds (243.94 ± 8.54 vs. 80.43 ± 8.92 mg CAE/g extract, respectively), as well as in achieving higher antioxidant capacity (ABTS: 78.28 ± 3.21 vs. 58.64 ± 2.46 mmol Trolox/g extract; DPPH: 69.29 ± 2.49 vs. 41.51 ± 2.06 mmol Trolox/g extract). The superior performance of TCE-UM is attributable to its ability to enhance cell disruption, leading to increased liberation of bioactive compounds.

The identification of key phenolic compounds, such as caffeic acid derivatives, flavonols, and flavan-3-ols, highlights the richness of the extracts in compounds associated with several health benefits. These phenolic compounds, particularly kaempferol and quercetin derivatives (e.g., kaempferol-3,7′-di-*O*-rhamnoside: 0.378 ± 0.025 µg/g), were significantly more abundant in TCE-UM extracts, which explains their enhanced antioxidant, enzyme-inhibitory, and antimicrobial properties. The study also highlights the multifunctionality of the extracts. TCE-UM demonstrated potent inhibition of skin-related enzymes, including tyrosinase (65.61 ± 5.21%), elastase (36.17 ± 6.26%), collagenase (26.17 ± 6.26%), and hyaluronidase (55.61 ± 5.39%), suggesting its potential application in anti-aging and skin-whitening formulations. The antimicrobial activity was particularly noteworthy against *S. aureus*, *E. coli*, and *C. albicans*, with the extract exhibiting concentration-dependent biofilm removal (up to 89.9 ± 4.9%) and metabolic inactivation. These findings underline the potential of TCE-UM extracts for addressing biofilm-associated infections and combating multidrug-resistant pathogens.

## Figures and Tables

**Table 1:** Identification and quantification of phenolic compounds in *Tamus communis* extracts via HPLC-DAD-MSn in negative mode.

Peak.	Rt (min)	λ max	[M − H]^−^ *m*/*z*	MS^2^	Tentative Identification	References	Concentration (mg mL^−1^)	
							TCE-CM	TCE-UM
**1**	3.79	306	341	179(100)	Caffeic acid hexoside	[11]	0.11 ± 0.00 b	0.31 ± 0.01 a
**2**	4.07	321	353	191(100),179(61),173(4),161(8),135(17)	3-*O*-Caffeoylquinic acid	[12]	0.23 ± 0.02 b	0.52 ± 0.08 a
**3**	5.27	313	369	223(100)	Sinapic acid rhamnoside	[13]	0.87 ± 0.02 b	1.17 ± 0.04 a
**4**	5.81	323	593	505(14),473(24),383(18),353(29),325(11)	Apigenin-6,8-*C*-diglucoside	[14]	0.90 ± 0.03 b	0.96 ± 0.02 a
**5**	7.96	348	609	301(100)	Quercetin-*O*-neohesperoside	[15]	0.46 ± 0.03 b	0.51 ± 0.02 a
**6**	10.34	280	351	163(100)	*p*-Coumaric acid derivative	[15]	0.67 ± 0.01 b	0.90 ± 0.10 a
**7**	11.84	346	593	431(21),285(100)	Kaempferol-3-*O*-neohesperidoside	[16]	0.06 ± 0.00 a	0.07 ± 0.01 a
**8**	12.14	335	563	431(10),285(33)	Kaempferol-*O*-rhamnosyl-*O*-pentoside	[17]	0.02 ± 0.00 b	0.03 ± 0.00 a
**9**	12.81	335	431	285(100)	Kaempferol-*O*-rhamnoside	[17]	0.03 ± 0.00 b	0.06 ± 0.00 a
**10**	13.52	354	463	301(100)	Quercetin-3-*O*-glucoside	[12]	0.01 ± 0.00 b	0.01 ± 0.00 a
**11**	14.98	343	577	431(100),285(50)	Kaempferol-3,7-di-*O*-rhamnoside	[17]	0.21 ± 0.00 b	0.38 ± 0.03 a
**12**	17.34	348	447	285(100)	Kaempferol-3-*O*-glucoside	[18]	tr	0.01 ± 0.00
					Total phenolic acids		1.88 ± 0.04	2.90 ± 0.24
					Total flavonoids		1.75 ± 0.07	2.12 ± 0.10
					Total phenolic compounds		3.64 ± 0.12	5.02 ± 0.34

Rt—Retention time; tr—traces. An independent samples t-Student test was conducted to compare the two extraction methods (TCE-CM vs. TCE-UM). Significant differences (*p* < 0.05) between the same compound under different extraction methods are indicated by different letters.

**Table 2:** Minimal inhibitory concentration and minimum bactericidal concentration (μg mL^−1^) of *Tamus communis* extracts.

	TCE-CM		TCE-UM	
ORGANISM	MIC	MBC	MIC	MBC
**Multidrug-resistant clinical isolates**				
*Staphylococcus aureus*	62.5	>62.5	31.25	>31.25
*Escherichia coli*	500	>500	125	>125
*Pseudomonas aeruginosa*	>1000	>1000	1000	>1000
*Klebsiella pneumoniae*	500	>500	62.5	>62.5
*Acinetobacter baumannii*	1000	>1000	500	>500
*Candida albicans*	250	250	62.5	62.5
**Foodborne isolates**				
*Salmonella typhi*	>1000	>1000	>1000	>1000
*Yersinia enterocolitica*	500	500	125	125
*Listeria monocytogenes*	>1000	>1000	1000	1000

## Data Availability

The original contributions presented in this study are included in the article. Further inquiries can be directed to the corresponding author(s).
